# Vulnerable Hours of the Day for Severe Iatrogenic Hypoglycaemia in Type 2 Diabetes: A Single-Centre Retrospective Study

**DOI:** 10.7759/cureus.61694

**Published:** 2024-06-04

**Authors:** Jun-Ichirou Mori, Koh Yamashita, Taku Yamashita, Masako Ueki, Shouichi Yoshiike, Toru Aizawa

**Affiliations:** 1 Center for Medical Education, Faculty of Medicine, Shinshu University, Matsumoto, JPN; 2 Diabetes Center, Aizawa Hospital, Matsumoto, JPN; 3 School of Pharmacy, Mukogawa Women's University, Nishinomiya, JPN; 4 Department of Obstetrics and Gynecology, Shinshu University, Matsumoto, JPN; 5 Department of Emergency and Critical Care Medicine, Aizawa Hospital, Matsumoto, JPN

**Keywords:** cosinor, oral hypoglycaemic agent, insulin, iatrogenic hypoglycaemia, type 2 diabetes mellitus

## Abstract

Introduction: Iatrogenic hypoglycaemia is an event that should be avoided in the treatment of diabetes, but the pathophysiology thereof has been poorly examined and reported. There is no established method for preventing iatrogenic hypoglycaemia and the current approach is a reactive response following onset of the disease. In this study, we aimed to explore the existence of ‘hypoglycaemia-vulnerable hours of the day’ in patients with type 2 diabetes, with the ultimate goal of preventing the onset of iatrogenic hypoglycaemia by clarifying the time when severe hypoglycaemia is likely to occur.

Methods: Of the 553,201 patients who visited the Critical Care and Emergency Center of Aizawa Hospital between 2008 and 2019, patients with proven hypoglycaemia (blood glucose level <3.0 mmol/L) and those using insulin or oral hypoglycaemic agents for the treatment of type 2 diabetes were included: 146 insulin users and 148 oral hypoglycaemic agent users. Cosinor analysis was employed to identify hypoglycaemia-vulnerable hours of the day.

Results: Patients with type 2 diabetes and severe hypoglycaemia had two peaks: at 8:00 and 18:00-19:00. Hypoglycaemia was observed as quadra-peaked in insulin users and double-peaked in oral hypoglycaemic agent users. Single-cosinor analysis revealed that the cycle was 5.83 hours (R=0.417) in insulin users, whereas it was 11.0 hours (R=0.717) in oral hypoglycaemic agent users. In insulin users, a significant periodicity of six hours (*P*=0.003) was observed in the cosinor detection analysis, and a significant correlation (*P*<0.05) was present in the cosinor percent rhythmicity analysis. In contrast, in oral hypoglycaemic agent users, a significant periodicity of 11 hours (*P*=0.03) was ascertained in the cosinor detection analysis, and there was a significant correlation (*P*<0.001) in the cosinor percent rhythmicity analysis. There were different hypoglycaemia-vulnerable hours of the day in the patients with type 2 diabetes, suggesting an interaction between disease pathophysiology and pharmacology.

Conclusions: These results can help elucidate the trend of the development of iatrogenic hypoglycaemia and contribute to the prevention of the onset thereof.

## Introduction

Diabetes mellitus is one of the most common non-communicable diseases in the world, with an increasing number of patients [1]. The control of hyperglycaemia and diabetic-related complications is the first priority in the treatment of diabetes [2,3]. However, treatment-related iatrogenic hypoglycaemia is often an obstacle to achieving strict glycaemic control [4-6]. To prevent iatrogenic hypoglycaemia, its pathophysiology should be fully understood; however, the current understanding of the pathophysiology of iatrogenic hypoglycaemia, especially circadian rhythmicity, remains insufficient. For example, in the Diabetes Control and Complications Trial/Epidemiology of Diabetes Interventions and Complications study, the incidence of hypoglycaemia increased when treatment was intensified; however, no further analysis on the vulnerable periods of the day has been conducted [3].

To the best of our knowledge, the likelihood of hypoglycaemia occurring at a specific time of the day is unknown. Such information is essential to minimise hypoglycaemia when intensive treatment is in place. Therefore, in this study, we aimed to explore ‘hypoglycaemia-vulnerable hours of the day’ (HVHD) in patients with diabetes using data from a large Japanese cohort.

## Materials and methods

Patient selection

Hypoglycaemia was defined as having a blood glucose level of <3.9 mmol/L, and severe hypoglycaemia was defined as having a blood glucose level of <3.0 mmol/L.

In this study, we defined patients with severe hypoglycaemia as those with pre-treatment blood glucose levels of <3.0 mmol/L and in whom recovery was difficult due to symptoms such as impaired consciousness; however, the symptoms in these patients could be improved by glucose administration. Further, patients with iatrogenic hypoglycaemia were defined as those with no recent history of heavy drinking, no obvious triggers such as weakness or a marked decrease in oral intake or an effect of other medication such as new quinolone antibiotics.

**Figure 1:**
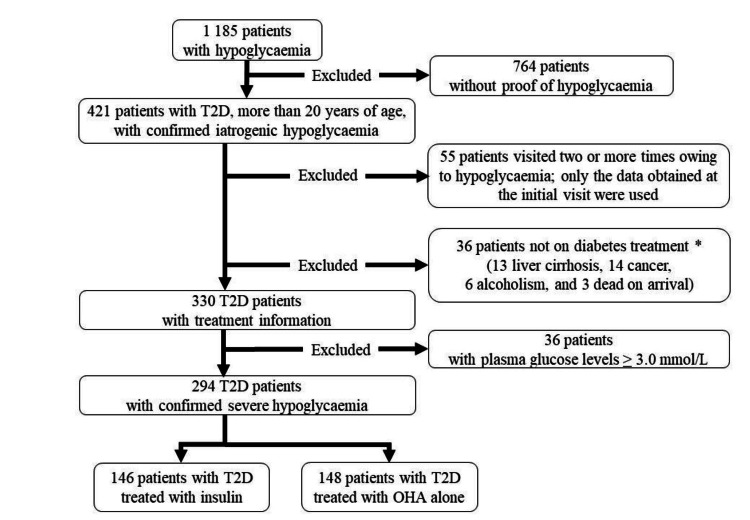
Patient selection protocol. T2D; type 2 diabetes mellitus, OHA; oral hypoglycaemic agent

Figure 1 shows the flow diagram of study participant selection in this medical record-based retrospective study. Initially, 1,185 patients with clinically suspected hypoglycaemia were selected from a total of 553,201 patients who visited the Aizawa Hospital Critical Care and Emergency Center from 1 January 2008 to 31 December 2019. Data on patients diagnosed with type 2 diabetes were extracted based on information from referral letters and medical history. However, 764 patients without type 2 diabetes, who did not have biochemical evidence of hypoglycaemia, or were under 20 years old, were excluded. For 55 patients who visited the emergency room (ER) two or more times owing to hypoglycaemia during the observation period, only the data obtained at the initial visit were used. Hypoglycaemia in patients with type 2 diabetes occurs owing to multiple causes: critical illness, alcohol use, cortisol deficiency, malnutrition, and others. In this study, 36 patients whose primary cause of hypoglycaemia was not insulin or oral hypoglycaemic agents (OHA) were excluded: this group of excluded patients had end-stage liver cirrhosis (n=13), advanced cancer (n=14), alcohol abuse (n=6), or cardiopulmonary arrest upon arrival (n=3). Among the 55 patients who visited the ER two or more times owing to hypoglycaemia during the observation period, we made the decision to include only data from the initial visit in our analysis of the HVHD. The choice was motivated by the concern that analysing multiple sets of data from the same patient might introduce bias related to individual-specific factors, such as lifestyle patterns, including eating behaviour and exercise habits. For this study, we included 330 patients with blood glucose levels below 3.9 mmol/L as patients with hypoglycaemia and 294 patients with blood glucose levels less than 3.0 mmol/L as patients with severe hypoglycaemia. They were classified into insulin users (Ins-users; n=146) and OHA users (n=148). The distinction was made based on the prescription given at the time of the ER visit owing to hypoglycaemia. The treatment of each patient (the type and dosing of insulin and OHA) was confirmed by patient interviews or referral letters to the attending physician.

This study was approved by the Ethics Review Committee of Aizawa Hospital Clinical Research (Approval date: January 5, 2021, Registry number of this study: #2020-057). Patients could opt out of the study through the hospital website and hospital postings.

Timing of hypoglycaemia onset

The time of onset of severe hypoglycaemia was defined as the time when the blood glucose level dropped and the patient required the assistance of another person to actively administer carbohydrates, glucagon, or take other corrective actions. However, many of the patients in this study exhibited impaired consciousness, and it was difficult to confirm the detailed time retrospectively. Therefore, in this study, the time of arrival at our ER was defined as the time of hypoglycaemia. There was a time lag between the onset of hypoglycaemia and the arrival at our hospital. In the Matsumoto area, the mean transfer time from the place where hypoglycaemia occurred to the ER was within 30 minutes; thus, arrival at the ER was assumed to be the time of hypoglycaemia onset for simplicity.

Physical and laboratory data

Physical and laboratory data were collected from electronic records. Since many of the patients usually went to other medical institutions and only visited our hospital in case of emergency, there were no detailed data on their history of drinking or diabetes. Furthermore, data on body weight and endogenous insulin secretion were missing in many patients.

Data analysis

Plasma glucose concentration was determined by the glucose oxidase method with Arkray Adams GA-1171 (Tokyo, Japan) or Labospect 008 Alpha (Hitachi, Ibaraki, Japan). Point-of-care glucose measurement was also utilised (Nova Biomedical Statstrip, Tokyo, Japan) [7]. Glycosylated haemoglobin A1c (HbA1c) levels were determined using Arkray HA8181 based on high-performance liquid chromatography. A biochemical analysis was performed using Labospect 008 Alpha.

The estimated glomerular filtration rate (eGFR) was determined by the following formula developed for Japanese people [8]: Males: eGFR = 194 × serum creatinine (mg/dL)^-1.094 × Age^-0.287; Females: eGFR = 194 × serum creatinine (mg/dL)^-1.094 × Age^-0.287 × 0.739.

Statistical analysis

Descriptive statistical analysis was performed by Wilcoxon rank sum, χ2, or Fisher’s exact test. HVHD was determined by time range analysis using the cosinor method [9]. Patient’s arrival at the ER was assumed to be the time of hypoglycaemia. JMP ver. 16 (SAS Institute, Cary, NC, USA), R ver. 4.2.2 (R Foundation for Statistical Computing, Vienna, Austria), and Kaleida Graph ver. 5 (Synergy Software, Reading, PA, USA) were used for statistical analysis.

## Results

Patient characteristics

Baseline data of Ins- and OHA-users are shown in Table 1. Among the 294 participants, 47 were receiving routine, regular treatment at our hospital: 29 Ins-users and 14 OHA-users. Only 12 were treated by board-certified diabetes specialists: nine Ins-users and three OHA-users. Therefore, most patients were receiving routine treatment outside our hospital.

**Table 1:** Characteristics of the study participants *P-value <0.05; **P-value <0.005 Ins group, patients treated with insulin; OHA group, patients treated with OHA only; IQR, interquartile range; SD, standard deviation; HbA1c, haemoglobin A1c; AST, aspartate transaminase; ALT, alanine transaminase; ALP, alkaline phosphatase; LDH, lactate dehydrogenase; GGT, gamma-glutamyl transferase; eGFR, estimated glomerular filtration rate; FIB-4 index = (AST × Age) / (Platelet × ALT^0.5); OHA, oral hypoglycaemic agent

Baseline characteristics	Total (n=294)	Ins group (n=146)	OHA group (n=148)	P-value
Male, number (%)	172 (58.5)	93 (63.7)	79 (53.4)	0.077
Age, years, median (IQR)	77 (71–83)	74 (67-80)	81 (75–85)	<0.001**
Transfer by ambulance, number (%)	251 (85.4)	123 (84.3)	128 (86.5)	0.623
Hospitalization, number (%)	137 (46.6)	45 (30.8)	92 (62.2)	<0.001**
Systolic blood pressure, median (IQR), (mmHg)	158 (141-178)	160 (140-180)	156 (141-174)	0.516
Diastolic blood pressure, median (IQR), (mmHg)	82 (69-91)	83 (69-92)	81 (70-92)	0.979
Heart rate, median (IQR), beats per minute	78 (68-90)	76 (67-90)	78 (69-90)	0.537
Plasma/blood glucose, median (IQR), (mg/dL)	32 (25-41)	32 (26-41)	32 (25-40)	0.860
HbA1c, median (IQR), (%)	6.6 (6.0–7.6)	7.6 (6.6–8.3)	6.2 (5.8–6.7)	<0.001**
AST, median (IQR), (IU/L)	28 (22–36)	26 (21–32)	30 (23–41)	0.002**
ALT, median (IQR), (IU/L)	17 (13–26)	17 (13–23)	19 (13–26)	0.483
ALP, median (IQR), (IU/L)	244 (198–294)	261 (217–320)	228 (186–281)	0.001**
LDH, median (IQR), (IU/L)	237 (202–281)	242 (209–280)	233 (198–283)	0.326
GGT, median (IQR), (IU/L)	19 (14–34)	20 (15–39)	18 (13–32)	0.044*
Serum creatinine, median (IQR), (μmol/L)	78.7 (61.0–130.8)	84.9 (61.9–147.6)	75.6 (60.1–130.2)	0.134
eGFR, median (IQR), (mL/min/1·73m^2^)	55.78 (31.43–75.29)	54.17 (30.35–75.39)	56.08 (33.93–73.78)	0.627
Red blood cell counts, median (IQR), (×10^4^/μL)	397 (354–438)	411 (371–453)	385 (345–430)	0.001**
Haemoglobin, median (IQR), (g/L)	121 (109–135)	124 (111–141)	119 (108–131)	0.013*
Haematocrit, median (IQR), (×10^-3^/L)	359 (326–397)	368 (333–415)	353 (324–388)	0.006*
c-reactive protein, median (IQR), (mg/L)	0.2 (0.1–0.3)	0.2 (0.0–0.5)	0.3 (0.1–1.3)	0.046*
FIB-4 index, median (IQR)	2.523 (1.844–3.651)	2.284 (1.673–3.230)	2.812 (2.063–4.095)	<0.001**

The median age was 74 and 81 years for Ins- and OHA-users, respectively; Ins-users were significantly younger than OHA-users (P<0.001). The number of Ins- and OHA-users transferred by ambulance was not significantly different: 123 (84.2%) and 128 (86.5%), respectively (P=0.623). Significantly fewer Ins- than OHA-users required hospitalisation owing to delayed recovery from hypoglycaemia (45 [30.8%] vs. 92 [62.2%], respectively; P<0.001). Blood pressure and pulse rate upon arrival were not significantly different between Ins- and OHA-users (median systolic blood pressure; 160 mmHg vs. 156 mmHg, P=0.516, median diastolic blood pressure; 83 mmHg vs. 81 mmHg, P=0.979, pulse rate; 76 beats per minute vs. 78 beats per minute, P=0.537, respectively).

The median plasma/blood glucose concentration upon arrival was 32 mg/dL in Ins-users and 32 mg/dL in OHA-users, with no significant difference between the two groups (P=0.860). The median HbA1c was significantly higher in Ins-users than in OHA-users (7.6% vs. 6.2%, P<0.001). For the evaluation of renal function, eGFR was also examined. The results showed an eGFR of 54.17 mL/min/1.73 m2 for Ins-users and 56.08 mL/min/1.73 m2 for OHA-users, which was not statistically significantly different (P=0.627).

Table 2 shows the results of the analysis of insulin treatment. The median dose of insulin in Ins-users was 22 units/day. The frequency of insulin injection was once a day in 41 patients (28.1%), twice a day in 47 patients (32.3%), three times a day in 34 patients (23.3%), four times a day in seven patients (4.8%), and uncertain in 17 patients (11.6%). The timing of insulin injection was morning in 117 patients (median dose of insulin=12.0 units), lunchtime in 48 patients (median dose of insulin=8.5 units), evening in 93 patients (median dose of insulin=10.0 units), and bedtime in seven patients (median dose of insulin=10.0 units). The median dose of each type of insulin was 12.6 units/day of rapid-acting insulin, 10.5 units/day of regular insulin, 16.8 units/day of neutral protamine Hagedorn (NPH) insulin, and 12.0 units/day of long-acting insulin. For morning users, the number of patients who used rapid-acting insulin was 45 (median dose, 6.0 units/day), those who used regular insulin was 41 (median dose; 6.0 units/day), those who used NPH insulin was 64 (median dose, 10.3 units/day), and those who used long-acting insulin was 23 (median dose, 14.9 units/day). At lunchtime, the number of patients who used rapid-acting insulin was 27 (median dose; 6.0 units/day), those who used regular insulin was 14 (median dose, 6.0 units/day), those who used NPH insulin was 13 (median dose, 5.6 units/day), and those who used long-acting insulin was seven (median dose, 12.0 units/day). In the evening, the number of patients who used rapid-acting insulin was 43 (median dose, 5.0 units/day), those who used regular insulin was 39 (median dose, 4.5 units/day), those who used NPH insulin was 61 (median dose, 6.0 units/day), and those who used long-acting insulin was seven (median dose, 10.0 units/day). At bedtime, no patients used rapid-acting insulin or regular insulin; the number of patients who used NPH insulin was one (median dose; 6.0 units/day), and those who used long-acting insulin were six (median dose; 15.0 units/day). The number of patients using premixed insulin was 57 in the morning, 13 at lunchtime, and 57 in the evening.

**Table 2:** Characteristics of the participants who were treated with insulin

The analysis of insulin treatment	Number	Median insulin dose (units)
Total daily insulin dose	-	22.0 (14.8–30.3)
Times of insulin injection	-	-
1	39	17.0 (10.0–23.0)
2	49	24.5 (18.0–35.8)
3	23	29.0 (18.0–36.0)
4	15	24.0 (20.0–36.0)
Unknown	20	-
Timing of insulin injection	-	-
Morning	117	12.0 (8.0–20.0)
Lunchtime	48	8.5 (6.0–12.0)
Evening	93	10.0 (6.0–14.0)
Bedtime	7	10.0 (5.0–20.0)
Type of insulin	-	-
Rapid-acting	45	12.6 (7.8–22.7)
Morning	45	6.0 (4.1–9.2)
Lunchtime	27	6.0 (4.0–9.0)
Evening	43	5.0 (3.0–8.0)
Bedtime	0	-
Regular	43	10.5 (6.0–18.0)
Morning	41	6.0 (3.6–10.0)
Lunchtime	14	6.0 (5.3–10.0)
Evening	39	4.5 (2.4–7.5)
Bedtime	0	-
Neutral Protamine Hagedorn	71	16.8 (10.5–22.0)
Morning	64	10.3 (7.0–14.5)
Lunchtime	13	5.6 (2.5–7.5)
Evening	61	6.0 (4.2–9.8)
Bedtime	1	5.0 (5.0–5.0)
Long-acting	42	12.0 (10.0–20.8)
Morning	23	14.0 (10.0–20.0)
Lunchtime	7	12.0 (10.0–28.0)
Evening	7	10.0 (6.0–20.0)
Bedtime	6	15.0 (5.3–23.0)
Pre-mixed insulin	62	26.0 (18.0–35.0)
Morning	56	15.5 (10.0–22.0)
Lunchtime	13	8.0 (5.0–14.0)
Evening	56	10.0 (6.0–15.0)
Bedtime	0	-

Analysis of the number of patients with hypoglycaemia each hour

**Figure 2:**
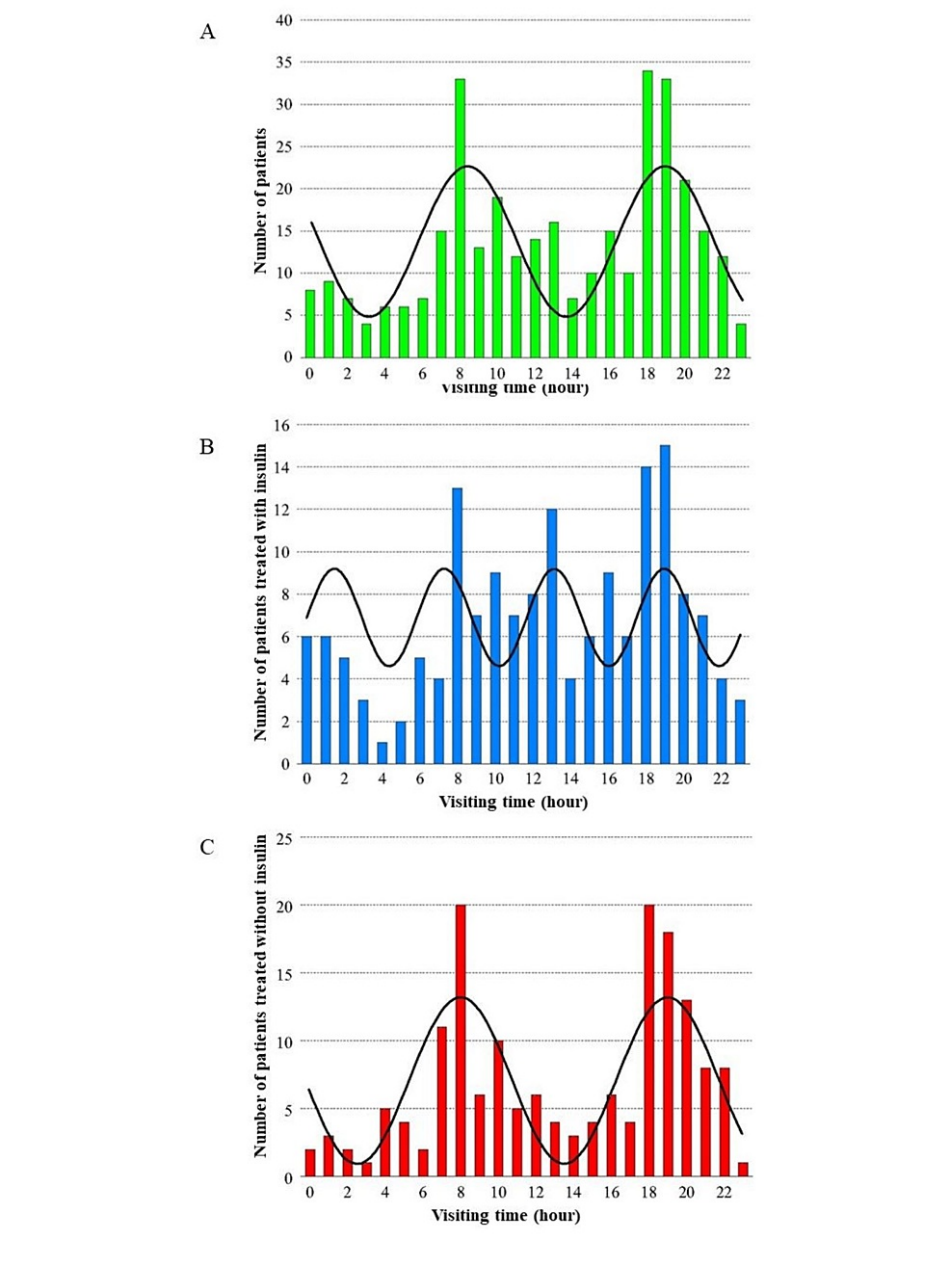
Analysis of the time zone when the patients in this study visited the emergency room for hypoglycaemia. The numbers of the patients are shown as bar graphs according to the visiting time (A, green), patients treated with insulin (B, blue), and patients without insulin (C, red). The numbers were fitted by the theoretical curves which were calculated by cosinor analysis in single-component models as shown by thick black lines. Note that the units on the y-axis differ between the three panels. A) Number of patients with hypoglycaemia. The number of patients in this study was determined according to data from the Critical Care and Emergency Center at Aizawa Hospital. Green: patients enrolled in this study. B) Number of patients with hypoglycaemia among patients treated with insulin. The number of patients who were treated with insulin was determined according to the time of visit to the Critical Care and Emergency Center at Aizawa Hospital. Blue: patients treated with insulin. C) Number of patients with hypoglycaemia among patients treated without insulin. The number of patients who were treated without insulin was determined according to the time of visit to the Critical Care and Emergency Center at Aizawa Hospital. Red: patients treated without insulin.

The time period when patients arrived at the ER for hypoglycaemia is displayed in Figure 2. Figure 2A shows the analysis of all patients. The number of patients who visited the ER for hypoglycaemia was highest at 08:00 and 18:00-19:00 hours. When Ins- and OHA-users were analysed separately, the unique pattern of HVHD was determined for each group (Figure 2B, 2C, respectively). Specifically, the number of patients who visited the ER for hypoglycaemia was lowest at 04:00 hours in Ins-users, whereas it was lowest at 03:00 and 23:00 hours in OHA-users. The cosinor method is one of the most common methods to analyse biological rhythm [10,11]. Single-cosinor analysis revealed that the cycle was 5.83 hours (R=0.417) in Ins-users and 11.00 hours (R=0.717) in OHA-users. In Ins-users, a significant periodicity of six hours (P<0.01) was observed in cosinor detection analysis, and a significant correlation was observed in cosinor percent rhythmicity analysis (P<0.05). A sub-analysis based on different periods showed that the above-described unique pattern of Ins-users and OHA-users was unaffected by time periods. In OHA-users, a significant periodicity of 11 hours (P=0.03) was observed in cosinor detection analysis, and a significant correlation was observed in cosinor percent rhythmicity analysis (P<0.001).

Next, the visiting time was divided into 12 time zones of two-hour intervals, and the factors associated with each time zone were examined. As factors, we examined the type of OHA, the timing of administration of OHA, the type of sulfonylurea (SU), the dosage level of SU, the type of insulin, the timing of injection, and the amount of insulin injected. As a result, there was no significant correlation among Ins-users for injection-related factors, such as insulin type, timing of injection, and dose level of insulin. Among the Ins-users, the number of patients with hypoglycaemia in the ~22:00-23:00 range was significantly higher than that of patients taking alpha-glucosidase inhibitor (αGI) who were not taking αGI (P=0.015). For OHA-users, the occurrence of hypoglycaemia was markedly reduced in patients using SU in the morning (P=0.003) during the ~00:00-01:00 range, significantly more in patients taking thiazolidinediones (TZD) (P=0.021) in the ~12:00-13:00 range (P=0.021), and significantly more in the ~20:00-21:00 range (P=0.038).

Furthermore, we examined the factors associated with the peaks observed in OHA-users, i.e., the time periods of 07:00-08:00 and 18:00-20:00. The number of patients in the 07:00-08:00 time period was higher in the group of patients who took SU in the evening and lower in the patients who injected insulin in the evening. In the analysis by type and timing of insulin, the number of patients who injected regular insulin in the morning and evening was small, and this trend was similar in patients who were injected with NPH insulin. Patients in the 07:00-08:00 time period were more likely to receive long-acting injections in the morning. In the 18:00-20:00 time period, it was more common in patients with a high fibrosis-4 (FIB-4) index and less in patients who were injected with regular insulin in the evening. In the Ins-users analysis, patients with the 07:00-08:00 time period were more likely to take SU in the evening and less likely to subcutaneously inject NPH insulin in the evening. It was also more common in patients using long-acting insulin, specifically in those injecting it in the morning. For the 07:00-08:00 time period, it was more common in patients who did not inject regular insulin in the evening. Notably, an analysis conducted on OHA-users revealed no dominant relationship between the type of SU, the amount of SU, and the timing of oral administration.

Medication

**Figure 3:**
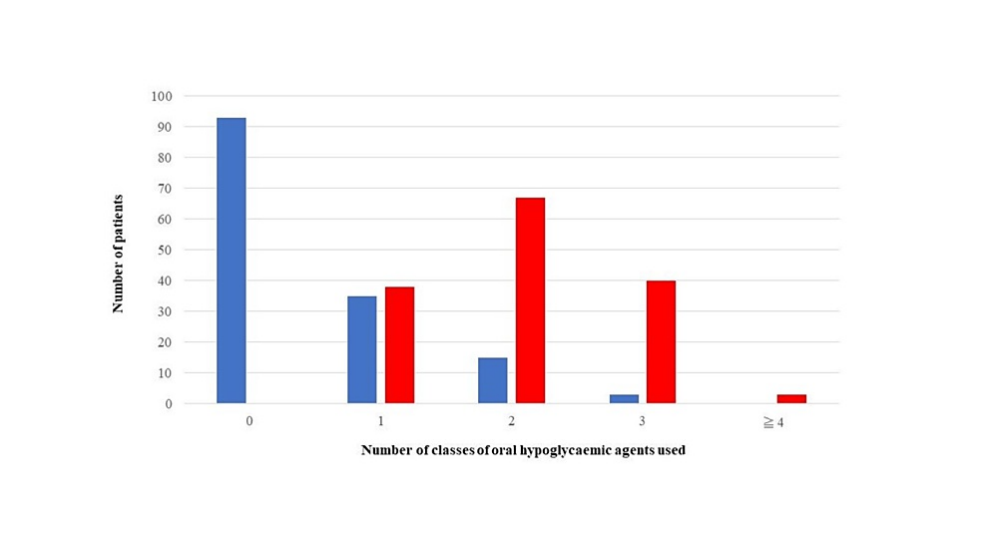
Types of oral hypoglycaemic agents. Most patients treated with insulin received treatment with insulin alone. In contrast, many patients not treated with insulin were administered two or more types of drugs. Blue: patients treated with insulin. Red: patients treated with oral hypoglycaemic agent. Group 0: insulin alone. Group 1: one class of oral hypoglycaemic agents. Group 2: two classes of oral hypoglycaemic agents. Group 3: three classes of oral hypoglycaemic agents. Group 4: four classes of oral hypoglycaemic agents. Group ≥5: five or more classes of oral hypoglycaemic agent.

Figure 3 shows the types of oral hypoglycaemic agents being taken by the patients. Among Ins-users, the majority of patients did not take any OHAs, with insulin alone being the predominant treatment. In contrast, in OHA-users, the number of patients who received one type of OHA was 38, 67 received two types of OHA, 40 received three types of OHA, and three received four types of OHA. As a drug with a high risk of hypoglycaemia, 144 patients were taking SU, and two were taking glinide; the other two patients were taking dipeptidyl peptidase 4 inhibitors (DPP4i) alone.

**Figure 4:**
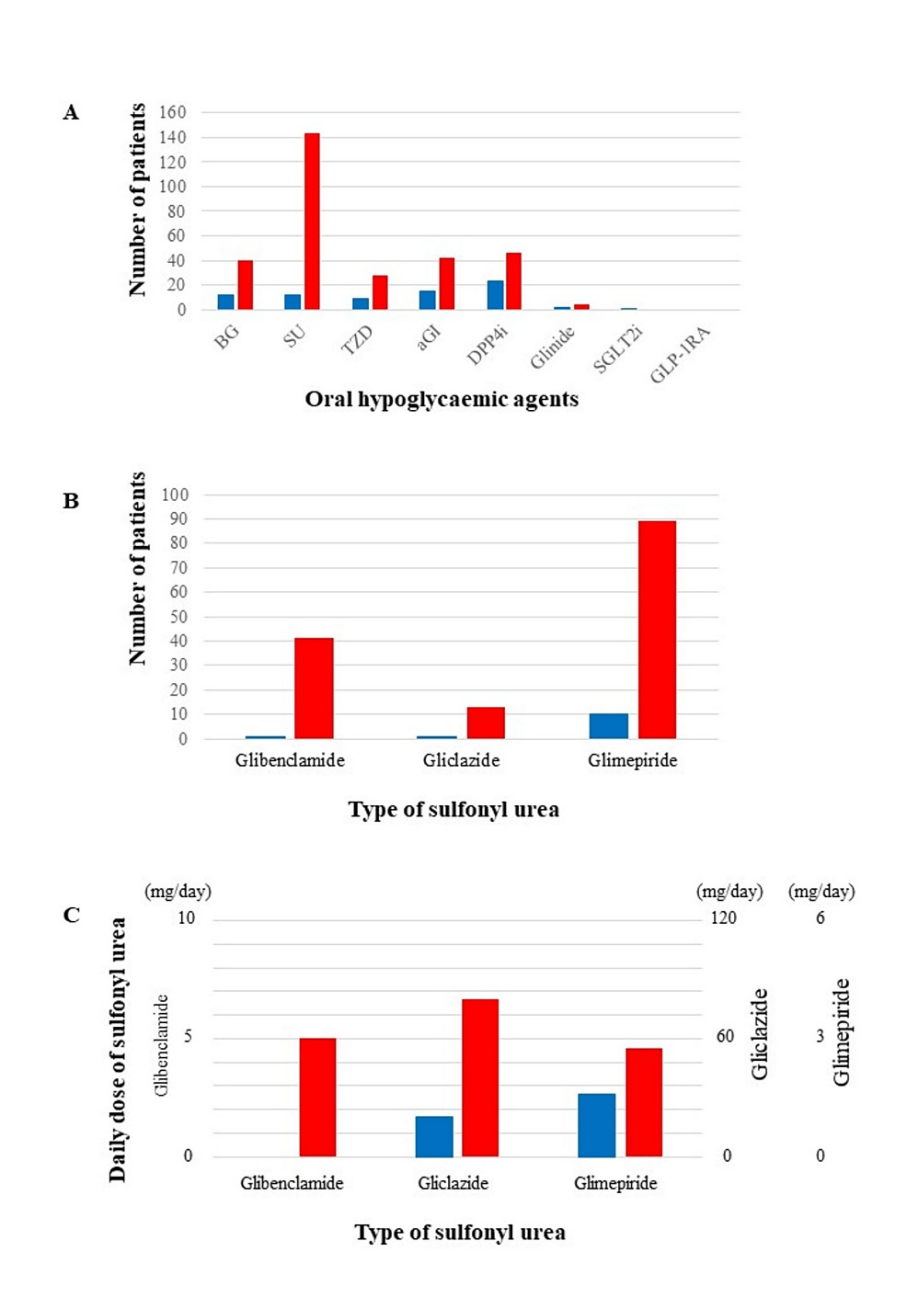
The details of the oral hypoglycaemic agents A; Number of patients taking each oral hypoglycaemic agent in both groups. In the group of patients treated with insulin, dipeptidyl peptidase-4 inhibitor (DPP4i) was the most used drug other than insulin, followed by alpha-glucosidase inhibitor (αGI) and biguanide (BG). In contrast, sulfonylurea (SU) was most common in the group of patients who were treated without insulin, followed by DPP4i and BG. Green: patients enrolled in this study; Blue: patients treated with insulin; Red: patients treated without insulin. B; Number of patients according to SU type. Type of SU administered to patients in each group: glimepiride was the most common, followed by glibenclamide and gliclazide. C; The average doses of each SU. Insulin users (Ins-users) took lower doses of any SU drug than oral hypoglycaemic agent users (OHA-users). In addition, among Ins-users, one patient used glibenclamide, but the dosage could not be confirmed. Blue: patients treated with insulin; Red: patients treated without insulin.

Figure 4 shows the details of OHAs. In this study, the patients were prescribed biguanide (BG), SU, TZD, αGI, insulin secretagogue (glinide), DPP4i, and sodium/glucose cotransporter-2 inhibitors (SGLT2i). No patient used glucagon-like peptide-1 receptor agonists (Figure 4A). Among Ins-users, many patients were taking DPP4i (n=23, 16%), whereas among OHA-users, almost all patients were taking SU (n=144, 97%). Significantly more OHA-users (n=144, 97%) than Ins-users (n=12, 8%) were taking SU (P<0.001), and significantly more OHA-users (n=40, 27%) than Ins-users (n=12, 8%) were taking BG (P<0.001). Similarly, significantly fewer Ins-users than OHA-users were taking TZD, αGI, or DPP4i (n=9 vs. 28, P<0.001; n=15 vs. 42, P<0.001; and n=23 vs. 46, P<0.01, respectively), but there was no significant difference between Ins-users and OHA-users regarding the number of people who were taking glinide and SGLT2i (n=2 vs. 4, P=0.69; and n=1 vs. 0, P=0.50, respectively). For SU, 123 patients took it in the morning, 15 patients took it at lunchtime, and 69 patients took it in the evening. By type of SU, 41 patients took glibenclamide, 13 patients took gliclazide, and 89 patients took glimepiride (Figure 4B). As for SU doses, Ins-users were prescribed lower doses compared to OHA-users (Figure 4C). The number of patients who received high doses of SU was 12 with glibenclamide (>5 mg/day), nine with gliclazide (>60 mg/day), and 20 with glimepiride (>3 mg/day).

## Discussion

In this study, we identified distinct peaks of iatrogenic hypoglycaemia during the 24-hour cycle in patients with type 2 diabetes receiving regular treatment. Specifically, there are certain points in time when hypoglycaemia occurs more frequently, for which we coined the term ‘HVHD’. The results of this study showed that the time of visiting the ER for severe hypoglycaemia peaked at 08:00 a.m. and 18:00-19:00, regardless of the treatment method. This result suggested that there are times when people are more likely to develop severe hypoglycaemia. The fact that the number of incidents of hypoglycaemia was higher during the day than at night was probably because it is easier to recognise hypoglycaemia during active moments, and family members are more likely to notice patients who are showing impaired consciousness due to hypoglycaemia. However, given that it was observed at a specific time of day rather than in equal abundance during daytime hours, it suggested the presence of HVHD. In addition, although the possibility of dietary effects cannot be ruled out, the effect was considered to be limited because no prominent peaks were observed at lunchtime.

Moreover, the pattern of hypoglycaemia was significantly different between Ins- and OHA-users: there were four peaks, six hours apart, and two peaks at 08:00 and 18:00-19:00, observed in the former and latter groups, respectively. Notably, the pattern of HVHD appears to be unaffected by the difference in time periods. This strongly indicates that it was independent of the type of OHA prescribed. The persistence of certain peaks in the two groups of patients indicated the involvement of some higher centres for rhythmicity. The food-entrainable oscillator is a complex circadian clock [12], and synchronisation by daytime-restricted food access has been observed in animal experiments [12,13].

We considered that the identification or recognition of HVHD would help reduce the degree and frequency of hypoglycaemia and mitigate hypoglycaemia-related adverse events during diabetes treatment. However, no comparable studies have been conducted.

Compared with Ins-users, OHA-users showed an increased need for hospitalisation, which was expected with the longer biological half-life of OHA compared with that of insulin preparations. Oscillations in plasma glucose levels of OHA-treated patients have been reported [14]. If such oscillation is generally present in patients with diabetes, the prediction of hypoglycaemia may be theoretically possible.

In both Ins- and OHA-users, details of the prescription, i.e., dosing, timing, and classes of hypoglycaemic agents, were highly variable, and we could not analyse subgroups on the basis of the prescriptions. Therefore, a pharmacokinetic explanation for the presence of peaks of hypoglycaemic events was not feasible. However, we can recommend that OHA-users should pay attention to daytime hypoglycaemia.

The strength of this study is the large, screened population included over a long period of time. Therefore, data were accumulated, and otherwise undetectable peaks were identified.

However, there are several limitations to this study. First, this was a retrospective, observational study from a single centre. This makes it difficult to identify a causal HVHD relationship, and the reproducibility of the results is unknown. Second, we could not gather detailed anthropometric data, such as body mass index and immunoreactive insulin, which could have helped to better understand the effect of these factors on HVHD/iatrogenic hypoglycaemia. Third, trends in pharmacology for diabetes changed during the study period [15]. Fourth, there was a difference in median age between OHA-users and Ins-users. We believe that differences in susceptibility to hypoglycaemia and treatment methods owing to age could result in bias. Fifth, as the surrogate of hypoglycemia onset, the time of the patient arrival at our hospital was taken. This is because it is officially reported that, in Matsumoto City, transfer of the emergency patient from the site to the hospital is achieved within 30 minutes. It is difficult to know exactly when hypoglycemia occurred, but the approximation we employed was acceptable if not scientifically correct.

## Conclusions

We identified distinct peaks of iatrogenic hypoglycaemia during the 24-hour cycle in patients with type 2 diabetes receiving regular treatment. Specifically, certain points in time when hypoglycaemia occurs more frequently were coined HVHD. We believe that this direction of research in clinical diabetes has the potential to provide a novel and powerful tool for achieving precise glycaemic control without inducing hypoglycaemia.
